# Medical Therapy of Acromegaly

**DOI:** 10.1155/2012/268957

**Published:** 2012-04-10

**Authors:** U. Plöckinger

**Affiliations:** Interdisziplinäres Stoffwechsel-Centrum, Charité-Universitätsmedizin Berlin, Campus Virchow-Klinikum, 13353 Berlin, Germany

## Abstract

This paper outlines the present status of medical therapy of acromegaly. Indications for permanent postoperative treatment, postirradiation treamtent to bridge the interval until remission as well as primary medical therapy are elaborated. Therapeutic efficacy of the different available drugs—somatostatin receptor ligands (SRLs), dopamine agonists, and the GH antagonist Pegvisomant—is discussed, as are the indications for and efficacy of their respective combinations. Information on their mechanism of action, and some pharmakokinetic data are included. Special emphasis is given to the difficulties to define remission criteria of acromegaly due to technical assay problems. An algorithm for medical therapy in acromegaly is provided.

## 1. Introduction

Surgical therapy of acromegaly aims for elimination of the tumour and normalisation of both, growth hormone (GH) secretion and Insulin-like growth factor-1 (IGF-1) concentration. Using conventional criteria for complete remission (GH below 1.0 *μ*g/L during oral 75-g glucose load (OGTT) and normal age- and sex-adjusted IGF-1 concentration) this will be achieved in about 60% of the patients. However, for patients with a macroadenoma (tumour diameter > 1 cm) and/or large parasellar extension, the rate of complete remission drops to 50% or less, even in experienced neurosurgical centres [1–6]. Freda et al. suggested very strict criteria for remission using a sensitive immunoradiometric assay (GH below 0.14 *μ*g/L during OGTT and normal IGF-1 concentration) [7]. However, for this criterion insufficient data are available to date. Of course, stricter criteria will result in fewer complete remissions.

Medical therapy is indicated in those acromegalic patients, who failed to achieve remission after surgery or in the rare patient with contraindication for surgical therapy. In addition, it is used in patients following radiotherapy in order to bridge the interval until complete remission.

In the following a short overview on the indications for medical therapy in acromegaly will be given. The different available forms of medical therapy will be treated in detail. Preoperative medical somatostatin analogue treatment will also be discussed. Finally, an algorithm for the medical treatment of acromegaly will be proposed.

## 2. Indication for Medical Therapy

A high GH- and IGF-1 concentration persisting after incomplete surgery or prevailing in the first years after irradiation is a clear indication for medical therapy. In contrast, in patients who just fail to reach the commonly accepted remission criteria, or even fulfil the biochemical criteria but still report discrete signs and symptoms of active acromegaly, the decision to initiate lifelong medical therapy may be difficult. Thus it is important to shortly review the problems of diagnosing persistent acromegaly in patients with a relatively low GH concentration after pituitary surgery and relating them to the commonly accepted remission criteria.

### 2.1. Remission Criteria

Cure of acromegaly as “restitutio ad integrum” is not possible, due to the irreversible bone changes. Thus, complete removal of the tumour, normalisation of the GH secretion and the IGF-1 concentration, as well as preservation of pituitary function, are generally aimed at. Criteria have been extensively discussed at different consensus conferences. The following criteria for complete remission are adopted by most centres [8]:

- a GH nadir below 1 *μ*g/L during an 75 g oral glucose load, GH determination at 30 minute intervals for at least 120 minutes [8–16],
- a normal age- and sex-adjusted IGF-1 concentration,
- no visible tumour tissue on MRI.

However, highly sensitive assays demonstrated active acromegaly with a GH nadir during OGTT well below 1 *μ*g/L. Freda et al. reported that five of fifteen newly diagnosed acromegalic patients reached a GH nadir during OGTT below 1 *μ*g/L, even as low as 0.42 *μ*g/L. The IGF-1 concentration was elevated in all patients, but signs and symptoms of acromegaly were subtle in three patients. Postoperatively acromegaly has been histologically confirmed in all these patients [17]. These data demonstrate the possibility of active acromegaly even with very low GH concentrations. Thus stricter criteria, using a sensitive monoclonal assay, have been proposed. These suggested criteria for remission refer to a GH nadir below 0.4 *μ*g/L [1, 8, 17–20] or even 0.14 *μ*g/L [7].

### 2.2. Evaluation of Remission Criteria

Whether failure to achieve these criteria for complete remission of acromegaly is identical to the indication for medical therapy is still a matter of debate. Most centres combine

- the results of biochemical investigations
- the clinical outcome of surgery, that is, does the patient still present signs and symptoms of acromegaly,
- the evaluation of the patient's quality of life, as recently has been suggested,
- epidemiological data, that is, the impact of an elevated GH and/or IGF-1 concentration on survival.

Thus a failure to achieve complete remission after surgery is by itself not necessarily an indication for medical therapy.

Moreover, the results for the nadir GH concentration during an OGGT and the IGF-1 concentration may be divergent or both tests may slightly fail to reach the predefined criteria. In such a case observation and reevaluation after, for example, 6 or 12 months is justified.

It has been proposed that patients with a mean GH concentration <2.5 *μ*g/L in a 5-point day profile need not be treated medically, since they have a life expectancy comparable to the normal population [21–23]. However, in a recent consensus discussion, this criterion failed to be seen as reliable and sufficiently evidenced based. The GH concentration determined by new and more sensitive assays is generally lower compared to the earlier used polyclonal assays. Thus using a random or mean GH concentration as the criterion for disease control, a GH concentration <1 *μ*g/L was suggested at an endocrine tumour summit [24].

The signs and symptoms of acromegaly are difficult to judge, when GH is low, although persistently autonomous. Reduced exercise capacity, lethargy, or sweating may be nonspecific. Follow-up and long-term observation of the quality of life (QoL) using an acromegaly adjusted questionnaire [25, 26] can be helpful in assessing the activity of autonomous GH secretion. Using the AcroQoL questionnaire Paisley et al. were able to show a negative correlation between well-being and the IGF-1 concentration, that is, higher QoL with IGF-1 concentration approaching normal values [27]. Bonapart et al. suggested that quality of life measurements may be able to uncover those patients who despite a normalisation of biochemical indicators still have active disease which possibly warrants further treatment [28]. Thus quality of life evaluation may offer additional clues with respect to treatment indications. Yet the significance of QoL questionnaire in the evaluation of acromegaly and/or treatment effects is still a matter of debate, as either negative, neutral, or positive effects of biochemical improvements have been demonstrated [29–31], or a positive impact has only been obvious for subscales on the QoL evaluation [32]. The lack of correlation between QoL and biochemical markers of the disease does not come as a surprise, as duration of the disease, extent of physical disfigurement, and comorbidities may have additional and individual impacts on the quality of life with acromegaly. Thus further and more detailed investigations will be needed, adjusting the results of QoL questionnaires to the aforementioned aspects, before QoL questionnaires will gain a major impact on the process of therapeutic decision making.

### 2.3. Technical Considerations

To establish the effect of surgical therapy several considerations have to be taken in account:

#### 2.3.1. Timing of the Postoperative Evaluation

Postoperative evaluation should be performed 3 to 6 months after surgery [8, 15, 33]. Earlier investigation, that is, an oral glucose load one week after surgery, has been claimed to be as reliable [8, 33]. However the IGF-1 concentration can take months to normalise and up to 30% of patients, especially those with discordant biochemical results, change their status within one year of follow-up. Hence evaluation after a 3 to 6 months interval is supposed to be the most reliable [15, 33–36].

The slow decline of the IGF-1 concentration is probably related to the high sensitivity of the liver after surgery, due to the now lower GH concentration. This may result in a delayed reduction of the IGF-binding-protein concentration and thus persistently increased IGF-1 concentration. The IGF-1 concentration may be elevated, despite an already normal GH suppression during OGTT, leading to divergent results. This can be observed in up to 30% of postoperative patients [16, 19]. The discrepancy can normalise over time, but it can also be related to minimal persistent autonomous GH secretion and thus indicate the possibility of relapse [15].

#### 2.3.2. Discrepancy between the GH Nadir during OGTT and the IGF-1 Concentration

The observed discrepancy between the GH nadir during an oral glucose load and the IGF-1 concentration is a major problem for the interpretation of biochemical indices of disease activity. The problem may be due to the difficulties in defining remission criteria for both, the GH-suppression by an oral glucose load and the normal IGF-1 concentration [8, 15–18, 20, 34, 35, 37–40].

This may be due to (i) altered dynamics of the GH secretion after surgery, (ii) inadequate GH nadir values (not adjusted to age, sex, and BMI), or (iii) to the influence of concomitant medication (i.e., SRLs, see below). In patients with radiotherapy the relation of GH to IGF-1 is disturbed and cannot be relied upon [41].

A divergence between the concentration of IGF-1 and the GH nadir during OGTT has been observed in both directions, that is, an increased IGF-1 and a normal GH nadir or a normal IGF-1 and insufficient suppression of the GH concentration [15, 42–44]. Both groups may demonstrate a change of their test results during long-term follow-up, indicating the difficulties with the definition of “complete remission” [45]. These uncertainties render the role of IGF-1 or the GH nadir during OGTT as the sole parameter for the estimation of prognosis (recurrence, morbidity, and mortality) of acromegaly problematic [10, 46, 47].

#### 2.3.3. Growth Hormone Assay

The various available assays for the determination of GH use different antibodies with distinct affinity to the GH molecule. These antibodies may recognise not only separate epitopes but even different GH isoforms. Most will recognize the 22 kD form of the GH molecule. Unfortunately the spectrum of isoforms recognised is rarely indicated. Furthermore the reference preparation used for the assay may differ between assays. Thus GH determination with different assays leads to divergent results. The difference between two assays for one GH sample can exceed 100% [48]. As the results of different assays are not comparable, each assay system needs its own reference value. This is important, since assay systems may change over time causing problems for the interpretation of results during long-term follow-up. Today most assays are sandwich chemiluminescence assays using a monoclonal antibody. These assays are very sensitive with a lower limit of detection of 0.002 *μ*g/L to 0.2 *μ*g/L.

Preanalytic handling is straight forward as GH, in a serum sample, remains stable for at least 24 hours [49]. In addition physiological variables such as age, sex, body mass index (BMI kg/m^2^), and the concentration of GH-binding protein (GHPB) may influence the GH concentration and the response to an oral glucose load. Generally GH concentrations are lower in older, obese, or male compared to younger, lean, or female subjects and patients [13]. So far, no data exist to adjust the nadir GH concentration during OGTT or the mean GH concentration during a GH profile for age, sex, and obesity [48]. This again highlights the difficulties in deciding on medical therapy based on the postoperative biochemical indicators in acromegaly [50].

#### 2.3.4. Insulin-Like Growth Factor-1

A normal IGF-1 concentration is not generally accepted as the only criterion for complete remission. In retrospective analyses of long-term outcome in acromegaly a normal IGF-1 concentration correlated positively with a normal survival of the patients [14, 23, 51, 52]. However there are valid arguments indicating that IGF-1 as the only determinant of remission may be insufficient. Postoperative normalisation of IGF-1 can take some time [53] (see above). False low values may be due to surgical stress or fasting, as both physiologically reduce the IGF-1 concentration. In addition numerous problems with the IGF-1 assay make IGF-1 an even less reliable parameter than the glucose-suppressed GH nadir. The possible pitfalls of the IGF-1 determination are either due to technical issues related to the assay system or to the reference preparation. In addition physiological variations of the IGF-1 concentration have to be taken into account.

#### 2.3.5. IGF-1 Assay

- Poor assay standardisation is one of the basic problems. The only available standard preparation (87/518) is impure, containing only 40% of IGF-1.
- IGF-binding proteins interfere with the antibody used to measure the IGF-1 plasma concentration. To reduce this interference several methods with varying efficacy are used. The “gold standard” is the addition of IGF-2. IGF-2 has a higher binding affinity for IGF-binding proteins than IGF-1. Adding IGF-2 during an ethanol extraction procedure reduces this kind of interference.
- The variability of the IGF-1 determination can be substantial. Two samples, measured at baseline and after two weeks, showed an overall good correlation (*r* = 0.922, *P* < 0.0001). However the reported differences ranged between −36.25% and +38.24% [54]. Intraindividual variability ranged from 3% to 36% [55].
- Recently it was demonstrated, that even two high quality assays, using the same international standard, were not comparable [42].

#### 2.3.6. Reference Populations and Physiological Variations

- The IGF-1 concentration is age dependent. Hence age-adjusted reference values have to be provided by each manufacturer. However most available assays provide insufficient data for reliable interpretation of the IGF-1 results [56]. Normative data have been published [57]. Unfortunately shortly thereafter, the assay system used (Nichols IGF-1 assay) was no longer available.
- In patients with poorly controlled diabetes mellitus or malnutrition IGFBP-1 and IGFBP-2 concentrations increase, rendering the determination of IGF-1 difficult [40].
- A number of clinical conditions will influence the IGF-1 concentration such as BMI (especially a BMI below 22 or above 37), ethnicity, chronic renal or hepatic failure, chronic undernutrition, and medication such as oral contraceptives [55, 58, 59].

The relative unreliability of the IGF-1 determination became even more a problem after the introduction of a GH antagonist, since the determination of the GH concentration is not meaningful during therapy [48, 60]. Details of medical therapy with a GH-antagonist will be discussed below. Thus, while the decision for treatment with a GH receptor antagonist may still rely on the nonsuppressibility of GH during OGTT, the follow-up during therapy and dose adjustment have to be based on a methodology demonstrated to lack sufficient sensitivity for the diagnosis of remission in acromegaly.

### 2.4. Conclusions

Indications for medical therapy are

- failure to sufficiently reduce GH secretion by surgery,
- bridging the time lag until complete remission will be achieved after irradiation,
- the rare patient with contraindication to surgery.

Recommended criteria for initiation of medical therapy are

- an insufficiently suppressed GH nadir (>1 *μ*g/L) during an oral glucose load,
- an increased age-adjusted IGF-1 concentration.

In patients with borderline results of the above discussed criteria decisions on the following considerations may support the decision for medical therapy,

- a mean GH concentration above 2.0 *μ*g/L–2.5 *μ*g/L during a five-point GH-profile,
- the clinical activity of acromegaly,
- the subjective activity of acromegaly investigated by the AcroQoL questionnaire.

Due to the difficulties in normative values for complete remission, the clinician is well advised to add clinical judgement and the severity of signs and symptoms of acromegaly into the decision algorithm for medical therapy [61].

## 3. Options for Medical Therapy

Medical therapy aims to achieve complete remission. Thus criteria already discussed will guide therapeutic decisions. Any deviation will be discussed when appropriate. The available drugs are long-acting somatostatin receptor ligands, dopamine agonists with low overall efficacy and the highly effective GH antagonist Pegvisomant.

### 3.1. Somatostatin Receptor Ligands (SRL)

**Mechanism of Action**

GH secretion is physiologically regulated by the interaction of stimulating and inhibiting hypothalamic peptides, growth hormone releasing hormone, and somatostatin, respectively. Somatostatin inhibits GH secretion from the pituitary somatotroph cells by binding to somatostatin receptors (sst). So far five different human somatostatin receptor subtypes, sst1, sst2A, sst3-5, are known. Binding of somatostatin to sst results in a G-protein-coupled activation of adenylate-cyclase. The intracellular signal transduction is dependent on the sst-subtype and the tissue involved and may include reduced Ca-influx, activation of phosphotyrosine-phosphatase, signal transduction pathways via STAT-5 or MAP-kinase, and activation of phospholipase A and C. Tentatively some specific roles may be related to each receptor: control of GH release via sst2 and sst5, modulation of insulin and glucagon secretion via sst5, induction of apoptosis via sst3 and possibly sst2, inhibition of the cell cycle via sst1 and sst3. Sst1 may play a role in angiogenesis, while the function of sst4 is still unknown. In comparison with normal pituitary tissue, somatotroph adenomas are characterized by a higher density of sst2 and sst5. The expression of sst in adenoma tissue is inhomogeneous and tumour specific. This may partly explain the variable responses of individual adenomas to SRL therapy [62].

Validating the presence of sst2 and sst5 on adenoma tissue has been suggested to allow the prediction of clinical efficacy of SRL. However immunohistochemical application for in vitro diagnostic has been hampered due to the lack of monoclonal and specific polyclonal anti-sst2 and sst5 antibodies. The use of two novel rabbit monoclonal anti-sst2A and anti-sst5 antibodies allowed for a several times more effective immunhistochemical staining for both, sst2A and sst5, respectively. Sst5 was seen in all, sst2 in 83% to 85% of the somatotropinomas. However, interindividual variability in the percentage of positive cells and the intensity of staining was pronounced [63–65]. Applying this specific and sensitive sst2A antibody we demonstrated that selective loss of sst2A correlated to Octreotide resistance in somatotroph adenomas [66]. Thus, using these highly sensitive tools may allow the prediction of an adenoma's response to SRL and even the selection of receptor-specific SRL.

On most tumours more than one sst subtype can be found. It has been shown that sst interact with each other or with other G-protein coupled receptors forming homo- or heterodimers, further increasing the complexity of somatostatin receptor activation. Receptor dimerisation may modify the receptor-ligand internalisation process, intracellular transduction signalling, and receptor recycling. Thus sst2A-sst3 heterodimerisation resulted in a new receptor with enhanced sst2A-like and diminished sst3-like activity [67]. Grant and Kumar demonstrated that sst2 and sst5 form heterodimers upon subtype-specific sst2 ligand activation [68]. This process leads to alterations in cell growth and may be responsible for the lack of tolerance seen in SRL treatment of pituitary tumours. Yet most of the specific mechanisms inducing receptor dimerisation, as well as the molecular consequences of this process, are still not clear. Further investigations are necessary to predict the effects of these many interactions on the decline of the GH concentration and inhibition of tumour growth.

Antiangiogenesis has been discussed as an additional mechanism of SRL. Somatostatin inhibits angiogenesis either directly or via growth factors, that is, vascular endothelial growth factor (VEGF), basic fibroblast growth factor (bFGF), and IGF-1 or through its immunomodulatory effects [69]. Recently it has been postulated that the antiangiogenic effects of SRL may affect tumour growth of pituitary adenomas. The extent of neovascularisation, as well as the expression of VEGF, correlates positively with the degree of invasiveness in some pituitary adenomas [69]. On the other hand pituitary tumours have been shown to be less vascular than the normal pituitary tissue [70]. This may explain the slow growth velocity of most pituitary adenomas. Interestingly in somatotropinomas microvessel density was negatively correlated with the patients' age, possibly explaining the clinical observation of more aggressive somatotroph adenomas in young compared to older adults [71–73]. Reduced angiogenesis in somatotroph adenomas may hamper the postulated antiangiogenic effect of SRLs on tumour growth. Using the chicken chorioallantoic membrane model Barrie et al. demonstrated the most pronounced antiangiogenetic effect by those SRL which preferentially activate sst2, like Octreotide or RC-160 [74]. Thus the presence of sst2, which is the dominant sst in most somatotroph adenomas, may be decisive for an antiangiogenetic effect by these drugs. For a detailed discussion of antiangiogenetic effects of SRL see Dasgupta [69].

**Somatostatin Receptor Ligands Octreotide and Lanreotide**

The clinical available somatostatin analogues Octreotide and Lanreotide demonstrate a high affinity for sst2 and a moderate affinity for sst5. The inhibition of GH secretion is mostly conveyed via sst2. However, as already discussed, receptor interactions modulated by subtype specific SRL may profoundly influence the mechanisms of action of SRL. In addition to the inhibition of GH secretion the native somatostatin, as well as the SRL, also reduce the secretion of insulin, glucagon, cholecystokinin and gastrin. For details on dosing and the duration of action see Table 1.

#### 3.1.1. Subcutaneous SRL Treatment

Octreotide and Lanreotide are cyclic peptides with a significantly prolonged half-life compared to somatostatin (2 hours versus 2 minutes). Only Octreotide is available for subcutaneous therapy. In monkeys Octreotide inhibits the GH secretion 45 times more than somatostatin [75]. Initially Octreotide was available for thrice daily subcutaneous injections. The doses used ranged from (50)–100 *μ*g up to 500 *μ*g three times a day. This regimen resulted in fluctuating drug concentrations, with an increase in GH usually observable six hours after the injection [76]. Octreotide (median 300 *μ*g/d) treatment of 189 acromegalic patients with a wide range of pretreatment GH concentrations and various forms of pretreatment resulted in a GH and IGF-1 concentration <5 *μ*g/L in 83/189 (45%) and <2 mU/L in 46/99 (46%) of the patients, respectively [77]. Comparable results were published by Ezzat et al. in a similar population of 152 acromegalic patients treated with two different dose regimens (300 *μ*g/d or 750 *μ*g/d). A GH concentration <5 *μ*g/L was achieved in 53% and 49%, the IGF-1 concentration normalized in 68% and 55% of the patients, respectively [78]. The now more stringent remission criteria, as well as changes in the IGF-1 determination, do not allow for a strict comparison with more recent results. Today subcutaneous therapy has been superseded by long-acting preparations. However, subcutaneous SRL treatment may still be indicated (i) in patients who need only very low doses of SRL to achieve remission criteria or (ii) in the rare patient with substantial hair loss during treatment with long-acting formulations, as this side effect may be less pronounced with the formulation for subcutaneous injections.

#### 3.1.2. Slow Release Formulations of SRL

The introduction of slow release formulations, Octreotide long-acting repeatable (LAR), Lanreotide slow release (SR), and Lanreotide Autogel makes possible an intramuscular or deep subcutaneous injection every 2–6 weeks (Table 1). The more constant plasma concentration of the long-acting formulation resulted in a slightly higher efficacy of Octreotide LAR versus subcutaneous therapy in 152 patients treated consecutively with both formulations [79]. Slow release formulations are more convenient for the patients and thus compliance is increased. Difficulties with injecting Octreotide LAR or Lanreotide Autogel can be reduced by delivering the injections with the help of specifically trained personnel.

**Therapeutic Efficacy of SRL**

SRL inhibit both, the physiological and the autonomous GH secretion. No tachyphylaxia has been observed during long-term therapy. This is in contrast to the declining efficacy in neuroendocrine gastrointestinal tumours treated with SRL. Treatment efficacy with SRL is assessed by using the basal or mean GH concentrations (<2.5 *μ*g/L) and/or normalisation of the IGF-1 concentration as already discussed. The GH nadir during an oral glucose load may not be helpful during SRL treatment for the assessment of therapeutic efficacy due to the high frequency of discordant results, that is, a normal IGF-1—but insufficiently suppressed GH concentration during OGTT. This may be due to (i) an SRL-induced direct suppression of the IGF-1 generation of the hepatocyte or (ii) a disturbance of the physiologic response of GH to oral glucose induced by SRL. In this case the failure to sufficiently suppress GH secretion by an oral glucose load is a treatment induced artefact [16] and this has to be kept in mind while interpreting published results. Octreotide LAR—and slightly less often Lanreotide SR—used as an adjunctive treatment after surgery, induces complete remission, defined by either a mean GH < 2.5 *μ*g/L and/or a GH nadir < 1 *μ*g/L during OGTT, in 56% and 49% of the patients, or a normalisation of the IGF-1 concentration in 66% and 48%, respectively. During primary therapy, remission criteria for GH or IGF-1 were achieved by both SRL in 50% or 60%, respectively [80]. Therapeutic results were negatively correlated to the basal GH concentration [80].

While Lanreotide SR has been shown to be slightly less effective than Octreotide LAR, a comparable efficacy has been demonstrated for Octreotide LAR and Lanreotide Autogel in two clinical trials [81, 82]. Schopohl et al. switched 37 patients on Octreotide LAR to Lanreotide Autogel. Both regimens achieved comparable results concerning the percentage of patients in remission as well as the mean GH (mean of a 5-point GH profile) or the IGF-1 concentration. In 50% of the patients the injection interval could be prolonged with Lanreotide Autogel, an advantage much appreciated by the patients [81].

In a further meta-analysis Freda confirmed their earlier results [80] and added follow-up information. The response rate increased over time [46]. Colao et al. reported their 5-years observational data in 45 de novo acromegalic patients. A surprising 100% and 98% control for the fasting GH (<2.5 *μ*g/L) and the IGF-1 concentration, respectively, was achieved. Again there was no difference between Octreotide LAR and Lanreotide Autogel [83]. The comparable efficacy of both, Octreotide LAR and Lanreotide Autogel has been further confirmed in a meta-analysis investigating the potency of different SRL formulations [84]. The clinical effects of SRL appear rapidly and often seem more pronounced than suggested by the biochemical results. Excellent effects are reported concerning headache, hyperhidrosis, soft tissue swelling, arthritic pain, and carpal-tunnel syndrome. Objective positive effects occur for the cardiovascular system (reduction of left ventricular hypertrophy, improvement of left ventricular ejection fraction) and hypertension [83, 85], prostrate (volume reduction) [86, 87], sleep-apnoea [88–90] and an improvement in renal structure and function [91].

**Side Effects**

Subcutaneous Octreotide injections can result in reddening of the skin and burning sensations at the injection site. This can be prevented by warming the drug to room temperature before injection (storage is normally in the refrigerator). With the depot preparation the injection is easily performed, if instructions for preparing the intramuscular injection are properly adhered to. Lanreotide Autogel can be self-injected by the patients.

All SRL induce nonspecific abdominal discomfort with nausea, flatulence, and loose stools during the first 3–5 days. These symptoms are mostly due to an inhibition of the exocrine pancreas secretion. In rare cases problems persist with steatorrhea and malabsorption. The addition of pancreatic enzymes resolves these problems.

The effect of SRL on glucose tolerance is not predictable. GH reduces the insulin sensitivity. The reduction of the GH concentration by SRL thus improves insulin sensitivity and hence the drug-induced inhibition of insulin secretion can be without negative effects on glucose tolerance. However in most patients SRL therapy slightly impairs glucose tolerance. Yet the clinical relevance is not clear. During an OGTT the first phase of insulin-secretion is reduced and the subsequent insulin increase is delayed.

However, the integrated insulin concentration is only slightly reduced [92]. Depending on the individual situation (obesity, metabolic syndrome, age) SRL can nevertheless induce diabetes mellitus. Patients with pre-existing diabetes mellitus may have an increased or reduced need of insulin treatment, and thus a tight control of blood sugar is mandatory in this group when SRL is initiated. Poor control of acromegaly predicted a deterioration of glucose tolerance during long-term treatment in patients on SRL therapy [76, 92–96].

SRL induce the inhibition of cholecystokinin secretion and the synthesis of a more vicious and lithogenic bile, resulting in gallbladder sludge or gallstones in up to 30% to 60% of the patients, respectively [97]. This complication occurs less often with the depot preparations. To dissolve gallbladder sludge, both chenodeoxycholic acid and ursodeoxycholic acid are highly efficient. Immediately after the conclusion of subcutaneous Octreotide therapy a short-lived hyperactive gallbladder contraction can be observed. This may give rise to biliary complications in patients with gallbladder sludge or gallstones. Probably due to the longer washout phase of depot preparations no such complications have been reported with the slow release formulations.

SRL reduce gastrin secretion. Together with a direct inhibitory effect at the parietal cell of the gastric mucosa, gastric acid—and probably intrinsic factor secretion—is reduced. Thus there is an increased risk of chronic gastritis and, with long-term therapy, a decline of the vitamin B12 concentration [97, 98]. This may be compensated by a monthly intramuscular injection of cyanocobalamin.

In rare cases an increase of liver function enzymes may occur. Thus control of these parameters is recommended every 6 months.

Diffuse alopecia is rare and occurs less often with the subcutaneous than with the slow release preparation and is reversible after discontinuation.

**Practical Issues**

Individually the result of SRL therapy is not predictable. A test dose of Octreotide (50 *μ*g or 100 *μ*g) injected subcutaneously followed by a GH-profile can have some prognostic value [99–101]. In contrast the positive and negative predictive values of GH suppression <2 *μ*g/L for long-term normalization of IGF 1 during Octreotide LAR therapy were 56% and 75%, respectively, in an investigation by de Herder et al. The authors concluded that the test cannot be recommended as a tool for clinical decision making on further therapy [102]. Overall the predictive value is limited as (i) the positive predictive value is higher for Octreotide LAR than Lanreotide SR [101], (ii) about 20% of patients with a negative test result may react with a GH decline during long-term medication, and (iii) the results are dependent on the criteria used to define remission.

The efficacy of the depot preparation should not be evaluated before steady-state drug concentrations have been achieved, that is, after 3 injections for Octreotide LAR.

Immunohistochemical determination of the sst2 may offer a better prognosis of the drug effects. As has been already mentioned, no tumour lacking sst2A immuno-histochemical positivity demonstrated an effect of SRL therapy [66].

Somatostatin scintigraphy does not correlate with the effects of SRL on GH [103].

In most cases a three-month intervention with a long-acting SRL will clarify the effectiveness of SRL therapy. With long-term therapy the SRL dose can be reduced in most patients. The determination of the GH and IGF-1 concentration should be performed immediately before the next injection, that is, at the nadir of the drug's plasma concentration.

#### 3.1.3. Preoperative SRL Treatment

Transsphenoidal surgery of a pituitary adenoma achieves complete remission in about 60% of the patients. The percentage may even be lower, when newer and stricter criteria of remission (GH nadir <0.14 *μ*g/L during OGTT) are taken into considerations. Surgical results correlate with tumour volume, especially so with supra- and parasellar adenomas, and with the preoperative GH concentration [1, 3, 10, 37, 52, 104–107]. Presurgical SRL therapy has been seen as a possible means to improve surgical outcome in patients with a macroadenoma.

**Tumour Shrinkage**

SRL reduce tumour volume in about 50% of previously untreated acromegalic patients. The extent of tumour shrinkage in these patients has been reported to be as high an 50%. However a critical meta-analysis by Melmed et al. showed overall significant tumour shrinkage—defined as at least 10% volume reduction—occurred only in up to 36.6% of therapy naive patients. The mean tumour volume reduction was 19.4% for the whole cohort, irrespective of whether individually significant shrinkage occurred [108]. This paper included definitions of tumour shrinkage over a wide range of volume reduction (from shrinkage of more than 2 mm, or 10% to 45%). There are some caveats with respect to these data. Firstly percentage reduction is of different significance whether calculated by the largest diameter or by volume reduction. Secondly it has been demonstrated that intra- and interindividual estimation of tumour volume by MRI may vary up to 20%. Thus any tumour volume reduction below 20% may be within the variability of the imaging method [109].

**Mechanism of Tumour Shrinkage**

The mechanism of tumour shrinkage is still not fully understood. SRL are not cytotoxic. Tumour shrinkage is probably in part due to an inhibition of the GH synthesis and subsequent volume reduction of the intracellular organelles involved in hormone production and intracellular transport. However, no consistent morphological changes have been observed [110–112].

Immunohistochemically SRL treatment induced a lower expression of Ki-67 staining, an indicator of dividing cells, compared to untreated somatotropinomas. The lower Ki-67 index indicates a suppressive effect of SRL on cell cycling [113]. Similar findings have been presented by Thapar et al. demonstrating a decreased growth fraction and an increased proportion of cells in G1 and M-phases. Thus the antiproliferative effects of SRL are probably related to an inhibition of cell growth, that is, reduced cell cycling rather than an increase in apoptosis [113, 114]. After discontinuation of SRL the process is reversible and tumour volume may increase within days [78, 115, 116].

**Practical Issues**

The duration of treatment necessary to induce significant tumour shrinkage is variable. In most responding patients tumour shrinkage can be observed within 3–6 months, while some may need SRL therapy up to one year to achieve significant volume reduction.

The analysis of factors predicting tumour response to SRL treatment does not show clear-cut correlations. Bevan, in a careful review, found contradictory reports with respect to the positive correlation of pretreatment tumour volume and response to SRL. Tumour shrinkage does occur in both, macro- and microadenomas [117]. Similarly, the GH or IGF-1 response to SRL as a prognostic factor for successful tumour shrinkage has been controversial. While some authors found that the decline of GH and/or IGF-1 concentration predicted tumour shrinkage in therapy naive patients, other could not demonstrate a correlation between biochemical efficacy and tumour shrinkage [117].

Volume reduction is most effective in preoperative, that is, therapy-naive patients. Tumour volume reduction is generally less in tumour remnants or after irradiation. In those patients tumour shrinkage has been reported in 27%, while shrinkage occurred in up to 52% in therapy-naïve patients [117]. Scarring and therapy-induced fibrosis may prevent significant tumour shrinkage in pretreated patients. SRL effects on tumour volume are independent of drug formulation. They have been observed with the subcutaneous injections of Octreotide as well as with the slow release preparations. In earlier publications increasing doses of s.c. injections were positively correlated to tumour shrinkage [78], while no such correlation was reported with the slow release preparations [118, 119]. This may be due to the more constant plasma concentrations of the drug with slow release formulations and a possible ceiling effect. Results of somatostatin-receptor scintigraphy do not predict tumour volume reduction [103].

**Effect of Preoperative SRL Treatment on Surgical Outcome**

Early publications suggested that presurgical therapy improves surgical outcome [120, 121]. However results of later reports were heterogeneous. An important improvement of surgical results in pretreated patients was reported by Colao et al. with a remission rate of 55% (*N* = 12) versus 30% (*N* = 11) in treated versus untreated patients, respectively [122]. A higher remission rate by presurgical SRL treatment was observed in patients with enclosed adenomas, but not in patients with invasive adenomas [123]. On the other hand Abe and Luedecke saw an improvement especially in invasive adenomas [124]. However, others could not confirm any advantage of presurgical SRL therapy, neither on short- or long-term postoperative comparisons [125–127].

Data from prospective randomized trials showed either (i) no statistical difference between patients pretreated or operated on without pretreatment (remission rate 55% and 69%, resp.) [128], (ii) a small, nonsignificant improvement (45% versus 23%, *P* = ns, pretreated versus primary surgery) [129], or (iii) a remarkable improvement (49% versus 18% *P* < 0.001, pretreated versus primary surgery) [130]. However, if only macroadenomas were analysed, Carlsen et al. found a significantly better surgical outcome for those patients pretreated with SRL (50% versus 16%) [129].

Thus it is still an open question whether presurgical therapy with subsequent tumour shrinkage really improves short- or long-term outcome of transsphenoidal surgery in acromegaly. It is possible, however, that in centres with relatively little surgical experience SRL pretreatment may improve surgical outcome, while results of very experienced centres cannot be further improved. Moreover, patients with a microadenoma will probably not profit from presurgical SRL treatment, while those with large macroadenomas may benefit [24, 61].

In addition to complete remission of autonomous GH secretion the preservation of pituitary function is an additional surgical goal. On this there is very little information. Our own investigation showed no positive effect of presurgical SRL treatment on the conservation of pituitary function [126].

Furthermore it has been shown that perioperative risk factors such as hypertension, poor cardiovascular-, pulmonary function, and diabetes mellitus may be positively influenced by preoperative SRL therapy. Reduction of soft tissue swelling occurring early in the time course of medical treatment may reduce difficulties of intubation. Thus presurgical therapy may be indicated in high-risk patients and should be discussed with the anaesthetist [122, 131, 132].

#### 3.1.4. Primary SRL Therapy

Primary medical therapy is defined as any medical therapy instead of surgery. Possible indications for primary medical therapy are (i) patient's preference, (ii) comorbidities that pose an anaesthetical or surgical risk, and (iii) possibly very old age [133]. In addition primary therapy may be discussed in patients with with an adenoma with large parasellar extension. In most of these cases surgery will fail to cure the patient. While surgery aims to remove all adenoma tissue, primary medical therapy tries to achieve biochemical remission, prevention of tumour growth, and possibly tumour volume reduction.

Several prospective nonrandomized trials as well as one retrospective analysis compared patients with primary and secondary SRL treatment. No significant difference of therapeutic efficacy was reported [119, 134, 135]. A publication from Baldelli et al. reported an even better result for primary therapy using Lanreotide SR [136]. Thus, in selected patients primary SRL treatment may offer an effective alternative to surgery. Importantly tumour growth during SRL therapy is very rare (Table 3).

However, there are some caveats: (i) none of these studies was randomized, (ii) in experienced hands the surgical risk is low, even in very old patients [137], and finally (iii) lifelong SRL treatment is very expensive.

#### 3.1.5. New Developments

New developments aim to find SRL with improved ligand-binding characteristics, as Pasireotide (Pasireotide), a multireceptor ligand or chimeric ligands binding to both, sst and dopamine D2 receptors.

**Somatostatin Multireceptor Ligands**

**Pasireotide (SOM 230)**

Pasireotide is an SRL with high affinity to sst-subtypes 1, 2, 3, and 5 [138, 139]. In comparison to Octreotide, Pasireotide has a longer half-life (2 hours versus 27 hours) [140]. This may be related to the intracellular dynamic of the sst2- and sst5-receptor-ligand complex. Both are internalised after ligand binding. However, sst5 is rapidly and extensively recycled from intracellular stores back into the cell membrane. Due to its long half-life, Pasireotide can bind again to the recycled sst5. Adenomas from patients resistant to the effect of Octreotide on GH secretion have been shown to express a high number of sst5. Thus Pasireotide may be especially effective in these patients. The high affinity of Pasireotide for sst1 and sst3 may prove to be an advantage for the stabilisation of tumour growth. These receptors are preferentially involved in growth inhibition. Both inhibit the progression of the cell cycle and induce apoptosis. A recently published study, compared Octreotide and Pasireotide (Octreotide 100 *μ*g s.c. thrice daily, followed by Pasireotide 200, 400 or 600 *μ*g s.c. twice daily for 28 days each) for the effect on GH, tumour growth, and glucose metabolism. The GH concentration was reduced to below 2.5 *μ*g/L in 9% and 19% of the patients for Octreotide and Pasireotide, respectively. In patients treated with Pasireotide for 3 months the response rate increased to 27%. Overall a reduction of the tumour volume of more than 20% was observed in 39% of the patients during treatment with Octreotide followed by Pasireotide. Side effects were comparable to those of Octreotide. However, the negative effect on glucose metabolism was more pronounced with Pasireotide [141].

**Somatoprim**

A recently developed somatostatin multi-receptor ligand, Somatoprim, may be at least as potent, as Octreotide, but without a negative effect on glucose metabolism. This molecule has a GH suppressing effect but a very low insulin-suppressing action in vitro [63, 142, 143]. If these in vitro data are confirmed in vivo in acromegalic patients, Somatoprim will be a valuable alternative to other SRL.

**Chimeric Molecules**

Another approach has been the development of “chimeric” molecules. These molecules bind to two different types of receptors, to the somatostatin- (subtypes 2 and 5) and the dopamine D2 receptor [144–146]. A substantial number of somatotroph adenomas express both sst and D2 receptors. Dopamine agonists inhibit GH secretion in some acromegalic patients and an additive suppressive effect on GH secretion can be demonstrated in some patients when dopamine agonists are added to SRL treatment. However, when preliminary in vitro results were followed by a “proof-of-principle” clinical trial the results failed to show any additional effect of this chimeric molecule on GH secretion.

**Conclusions**

SRL are the first-line treatment in patients with insufficiently controlled GH secretion after surgery. The slow release formulations Octreotide LAR and Lanreotide Autogel are equally effective. Up to 60% of the patients will achieve complete remission and the rate increases with prolonged treatment. Primary treatment with SRL should be reserved for those rare patients who refuse or have contraindications to surgery. Primary medical therapy may achieve tumour stabilisation in most patients. Efficacy of primary medical therapy may be comparable to postoperative medical therapy. With long-term therapy a dose reduction may be possible. Long-acting SRL are well tolerated. Preoperative SRL therapy offers no advantage in microadenomas, but may be indicated in large macroadenomas to reduce tumour volume and/or perioperative complications.

### 3.2. Dopamine Agonists (DAs)

Dopamine agonists suppress GH secretion only modestly in most patients and have largely been replaced by somatostatin receptor ligands. However, they are much cheaper than SRL or Pegvisomant and can be taken orally. At present they are mainly used as an adjunct to insufficient SRL therapy.

In healthy man dopamine agonists stimulate GH secretion, while in acromegalic patients GH secretion is paradoxically inhibited. In 1972 Liuzzi et al. detected the paradox GH-reducing effect of L-Dopa in patients with acromegaly [147]. This resulted in the development of the “first-generation” dopamine agonist preparation, Bromocriptine, and was soon followed by “second-generation” dopamine agonists such as Cabergoline and Quinagolide (a nonergot dopamine agonist). These have a longer half-life and fewer side effects.

DAs bind preferentially with high affinity to dopamine 2 (D2) receptors. Due to a high first-pass effect in the liver only 6% of a given dose will appear in the peripheral circulation. Elimination of the drug is by hepatic metabolism and excretion via the bile. Thus, with reduced liver function a dose reduction is recommended. Gastrointestinal side-effects of bromocriptine (nausea, vomiting, abdominal discomfort, constipation, or diarrhoea) can be reduced by a very slow dose increase. First-generation DA—Bromocriptine, Lisurid and Methysergid—are now rarely used in acromegalic patients. The second generation DAs have to be taken daily (Quinagolide) or once to twice weekly (Cabergoline). Increasing the dose to more than the recommended maximal dose does not increase efficacy, but results in worse side effects. DAs have been reported to be more effective in adenomas that co-secrete prolactin (PRL), which express more D2 receptors [148], but this has not been universally confirmed [149]. It would, however, be compatible with the good response to DA treatment in monohormonal prolactinomas.

**Cabergoline**

Cabergoline is more effective than Bromocriptine, Methysergid, or Lisurid. The plasma half-life of Cabergoline is between 62 and 115 hours. With weekly intake, steady state is achieved after approximately 4 weeks. With a dose of 1.75 mg/week to 2.75 mg/week the GH concentration declined to below 2 *μ*g/L in 46% of the patients and the IGF-1 concentration was reduced to below 300 *μ*g/L in 39% [150]. The excellent results of this publication may be attributable to a high number of patients with mamma-somatroph or mixed lacto-somatroph adenomas. In a Belgian multicentre trial with 64 acromegalic patients receiving Cabergoline 1–1.75 mg/d once weekly, the IGF-1 concentration declined to below 300 *μ*g/L in 8 of 16 (50%) patients with GH/PRL cosecretion and in 39% of the whole group. The GH concentration declined to below 2 *μ*g/L in 46% of all patients [148]. Similar responses (normal IGF-1 concentration 34%, GH < 2.5 *μ*g/L 48%, resp.) are reported in a meta-analysis of nine studies representing 149 patients [151]. In contrast Freda and coworkers found a persistently normal IGF-1 concentration in only 3 of 14 (21%) patients, without a preferential effect in patients with hyperprolactinemia [149]. Overall the effect of Cabergoline correlates with the initial GH/IGF-1 concentration, that is, the lower the initial GH/IGF-1 concentration the higher the probability of a significant decline or normalisation of the GH and IGF-1 concentration, respectively.

Tumour shrinkage occurred in 13 of 21 patients, with a mass reduction by more than half in 5 GH-/PRL co-secreting adenomas [148]. In five studies that prospectively investigated tumour shrinkage during Cabergoline therapy tumour shrinkage was associated with a higher baseline PRL and IGF-1 concentration [151].

**Quinagolide**

Quinagolide is a nonergot selective D2 agonist. Half-life during steady state is about 17 hours. There are relatively few data on the effect of Quinagolide in acromegaly [152–156]. After a single 150 *μ*g dose of Quinagolide the GH concentration decreased by 49% in ten acromegalic patients [154]. Lombardi et al. treated 12 patients resistant to SRL therapy with Quinagolide 150 *μ*g/d in a prospective, placebo-controlled trial. In 33% of the patients the GH concentration declined from 35 *μ*g/L to 2.7 *μ*g/L. Increasing the dose to 300 *μ*g/d added one more responder. The seven patients unresponsive to Quinagolide were subjected to a combination therapy of Octreotide (600 *μ*g/d), and Quinagolide (600 *μ*g/d) for three months. In two of these combined therapy induced a greater GH reduction than either medication alone [156]. Colao et al. compared the effect of Bromocriptine, Cabergoline, and Quinagolide in 34 acromegalic patients. They concluded that both, Cabergoline and Bromocriptine (given as a LAR preparation), cannot be considered useful medical approaches for acromegaly, whereas Quinagolide normalised circulating GH and IGF-I levels in 47.8% of patients [150]. However, normalisation was defined as a GH concentration below 5 *μ*g/L and the IGF-1 values were not age-adjusted.

**Conclusions**

Monotherapy with a dopamine agonists should be considered as first-line treatment in patients with relatively low GH and IGF-I concentrations in view of their low cost and oral intake. They may be more efficient in patients whose adenoma co-secretes prolactin.

**Combination Therapy of SRL and Dopamine Agonists**

In patients insufficiently responding to SRL or dopamine agonist treatment, combination therapy may be an option to further decrease GH secretion [151, 157–163]. Marzullo et al. investigated the effect of a 6-month treatment with Lanreotide (60–90 mg/month), alone and in combination with Cabergoline 1.5–3 mg/week or Quinagolide (0.6 mg/day) in 10 acromegalic patients. The addition of dopamine agonists resulted in a further decline of the GH and IGF-1 concentration not achieved by SRL treatment alone [158]. Minniti et al. studied the acute effect of the combination of Octreotide s.c. and Cabergoline in 21 patients with acromegaly. In 14 patients with insufficient response to either drug, combination therapy was initiated (Octreotide 100 *μ*g s.c. plus Cabergoline 0.5 mg p.o. 24 h before Octreotide) resulting in a significant further GH decline [157]. A more recent investigation combing SRL slow release formulations with Cabergoline (1–3.5 mg/week) in 19 acromegalic patients decreased GH to <2.5 *μ*g/L in four (21%) and normalised the age-adjusted IGF-1 concentration in eight patients (42%). The effect was independent of the cosecretion of PRL [159]. With IGF-1 concentration as an endpoint, normalisation was observed in 19 patients (56%) with already relatively low IGF-I concentration on Octreotide LAR monotherapy. The effect was independent of the baseline PRL concentration or GH/PRL co-expression [162, 163]. In a meta-analysis Sandret et al. confirmed the usefulness of adding Cabergoline to SRL in patients with an insufficient effect of SRL therapy. Cabergoline adjunction normalised IGF-I in about 50% of cases. The change in the IGF-I concentration was significantly related to the baseline IGF-I concentration but not to the dose of Cabergoline, the duration of treatment, or the baseline prolactin concentration [151].

**Side-Effects**

Side-effects of dopamine agonist are given in Table 2. They increase with the dose and can be prevented by a slow increase of the dose. Nausea and orthostatic dysregulation can be precluded by taking the dopamine agonist at bedtime. Vasospastic adverse effects require a dose reduction. Side-effects occur less often with the second generation DA. However DA may induce a psychosis and this can occur even after long-time exposure. Since a patient may not notice the problem, relatives should be informed of this possible side-effect. Pre existing psychiatric illness is a relative contraindication to DA therapy. High dose Cabergoline therapy has been reported to cause cardiac valvular abnormalities. With long-term, high dose Cabergoline therapy echocardiographic follow-up has been recommended [61, 164, 165].

**Conclusions**

**Figure 1:**
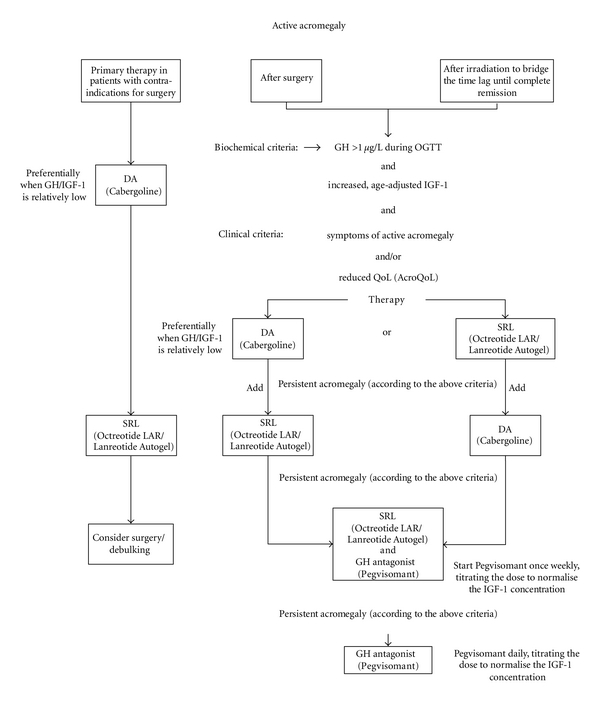
Therapeutic algorithm.

Dopamine agonist drugs may be used after incomplete surgery in acromegalic patients. This is mainly recommended in patients with only modestly elevated GH/IGF-1 concentrations. Whether patients with PRL cosecretion will have a greater benefit remains unclear. Cabergoline is most often used. DA monotherapy results in complete remission in less than one third of the patients. Patients with insufficient GH/IGF-1 suppression during SRL monotherapy may benefit from addition of a dopamine agonist, which will further lower GH/IGF-1 concentration in up to 50%. This combination is substantially cheaper than the addition of a GH-antagonist and should therefore be tried before adding Pegvisomant (Figure 1).

### 3.3. Growth-Hormone Receptor-Antagonist

Following incomplete surgery established medical therapies with SRL, dopamine agonists or a combination of both successfully reduce GH secretion in up to 75–80% of the patients. In patients who fail to achieve complete remission growth-hormone receptor-antagonist (GHA) therapy is an option.

**Mechanism of Action**

Pegvisomant, the only available GHA, is an analogue of the 22 kD GH molecule. Modification of the GH molecule, exchanging the amino acid glycin at position 120 in the third helix, results in a GH antagonistic effect. Further modification, an exchange of eight amino acids at the binding site-1, was thought to increase the affinity to the GH-receptor. However, it has been demonstrated that this exchange does not affect affinity but removes two sites for polyethylene glycol (PEG) binding, which are within the native binding site [166, 167]. Thus modified, binding to the receptor is reduced, requiring high plasma concentrations of the GHA to competitively block the activation of the receptor. Pegylation of the molecule prolongs the plasma half-life from 15 minutes to 72 hours [168, 169]. Furthermore pegylation reduces immunogenicity of the molecule as well as interaction with GH-binding proteins [167]. With monotherapy the drug has to be injected subcutaneously on a daily basis. Alternate day injections of Pegvisomant failed to maintain IGF-1 within the age-adjusted range [170].

Peak serum Pegvisomant concentrations are attained between 33–77 hours after administration. The half-life is approximately 6 days, which is 6 times longer than the daily 24-h dosing interval in clinical use. Thus peak-trough fluctuations at steady state during once daily dosing are small [171]. Pegvisomant circulates at concentrations 100–1000 times those of endogenous GH. An important question was if Pegvisomant can cross the blood brain barrier via putative choroid-plexus GH receptors and thus interacting with GH-receptors at the hippocampus, cerebral cortex, and hypothalamus, possibly influencing mood, cortical blood flow, and neuronal growth. Veldhuis et al. demonstrated, based upon CSF measurements, that the pegylated GH-receptor antagonist does not cross the human blood-brain barrier [172].

**Practical Issues**

Due to the similarity between the structure of GH and Pegvisomant most available GH assays crossreact with Pegvisomant. Furthermore GH-receptor blockade induces a feedback increase of the endogenous GH concentration. Therefore the determination of GH can no longer be used as a parameter for therapeutic efficacy. Hence normalisation of the IGF-1 concentration is the therapeutic goal. The determination of the IGF-1 concentration is used for follow-up during therapy and dose adjustment. Problems of the IGF-1 assay have already been discussed.

Further challenges for the evaluation of therapeutic efficacy refer to the concept of a dose-dependent tissue-specific effect of Pegvisomant. Adipose tissue, the kidneys, and skeletal muscle seem to need less Pegvisomant to reduce GH actions compared to the liver where more Pegvisomant is required in order to reduce IGF-1 production [173]. During Pegvisomant therapy normalisation of hepatic IGF-1 synthesis may thus be accompanied by a status of peripheral functional GH deficiency despite a normal IGF-1 concentration. We found increased visceral obesity but no improvement of glucose tolerance in 5 patients after 6 months of Pegvisomant therapy resulting in normal IGF-1 concentrations. This combination supposedly indicates peripheral GH deficiency with reduced lipolysis and a subsequent increase in intra-abdominal fat mass, as well as increased insulin resistance of the liver and muscle [174, 175]. These data reinforce the discussion about the concept of “extra-hepatic acromegaly” [173] and the resulting need for new biomarkers for the evaluation of therapeutic efficacy in acromegaly.

**Therapeutic Efficacy**

Pegvisomant treatment results in normalisation of the IGF-1 concentration in 90% to 97% of patients with up to a maximal doses of 40 mg/d s.c. or a median dose of Pegvisomant of 130 mg/week [176, 177]. The dose of Pegvisomant needed for normalisation of the IGF-1 concentration is positively correlated to the basal age- and sex-adjusted IGF-1 concentration and is independent of pretreatment modalities (primary medical therapy versus postoperative therapy or combination therapy) [178]. Recently Ghigo et al. compared the efficacy of Pegvisomant and Octreotide LAR in an open randomized multicentre study in 152 acromegalic patients. The normalisation rate of IGF-1 was similar in both groups, but Pegvisomant was more effective in those patients with higher IGF-1 baseline levels [179]. “Real world” data collected in the “Acrostudy,” a large observational survey, demonstrated normalisation of the IGF-1 concentration in only 60%–70% of the patients. These results were possibly due to insufficient dose titration of Pegvisomant [180].

**Combination Therapy of Pegvisomant and SRL**

In consideration of the high costs of Pegvisomant therapy Feenstra et al. investigated the effect of monthly Octreotide LAR treatment in combination with Pegvisomant injected once weekly with a dose of up to 80 mg/week [178]. In patients insufficiently treated with Octreotide LAR, normalisation was achieved in 95%, on a median dose of 60 mg (range 40–80 mg) Pegvisomant per week. In contrast, in another study referred to by the authors, this dose, given as monotherapy, resulted in normalisation of the IGF-1 concentration only in about one third of the patients. Thus, combined treatment allowed for a dose reduction of Pegvisomant compared to the median 130 mg/week dose used in the long-term monotherapy trial [177]. Possibly less Pegvisomant is needed when, due to the effect of SRL, less growth hormone is to compete with in the circulation. In addition, the reduction of insulin in the portal vein during SRL therapy will decrease the number of available GH-receptors and thus increase the efficacy of GH receptor blockade by Pegvisomant [178]. While combination therapy results in a significant reduction of the weekly Pegvisomant dose compared to monotherapy, interindividual variability is high. This may be due the exon-3 polymorphism of the human GH-receptor gene. 40%–50% of the Western population carries an exon-3 deleted GH-receptor. Pegvisomant therapy was more effective in those acromegalic patients positive for the exon-3 deletion of the GH-receptor. A 20% lower dose per kg bodyweight achieved normalisation of the IGF-1 concentration compared to patients with the full-length receptor variant [181]. The higher the dose of Pegvisomant in patients on monotherapy, the higher is the probability of saving up to 50% of the dose with concomitant SRL therapy. Thus combination therapy is also more cost efficient than monotherapy.

**Clinical Efficacy**

In addition to assessing the biochemical effects of Pegvisomant therapy a small number of trials focused on specific endpoints.

**Cardiovascular Endpoints**

Pivonello et al. reported a significant reduction of left ventricular hypertrophy and improved diastolic and systolic performance [182]. An additional study showed a significant reduction of the diastolic blood pressure but no effect on systolic blood pressure in four hypertensive patients on antihypertensive medication during Pegvisomant therapy [183]. However, overall cardiovascular improvement is probably less than with SRL, due to a possible additional and direct effect of SRL on cardiac tissue [184].

Pegvisomant therapy resulted in a reduction of the tongue volume and this was accompanied by a decrease of the apnoea-hypopnoea index in patients with sleep-apnoea. In this small study the decline of both parameters did not correlate with the decline of the IGF-1 concentration [185]. The brachial artery vascular function, which is known to be decreased in active acromegaly, improved in 10 patients treated with 10–40 mg/d Pegvisomant for 18 months [186].

**Metabolic Effects**

During short-term (4 weeks) administration of Pegvisomant the IGF-1 concentration (normalisation of the IGF-1 concentration in four of the five patients), and the insulin concentration declined. Insulin sensitivity of basal lipolysis and endogenous glucose production were enhanced compared to baseline [187]. The fasting glucose- and insulin concentration, as well as the homeostasis model assessment index (HOMA), an estimate of insulin resistance, declined during Pegvisomant compared to baseline. While total cholesterol concentration remained unchanged, HDL cholesterol increased significantly in patients on a daily Pegvisomant dose ranging from 10 to 40 mg/d during a treatment period of 12 months. Serum IGF-1 had normalised in 75% of the patients, but there was no correlation to the observed metabolic changes [183].

**Bone Formation**

Patients with acromegaly tend to have normal or even slightly increased bone mineral density. However both, markers of bone formation and resorption, have been found to be increased, as was the risk for vertebral fractures. Jimenez et al. showed in a small heterogeneous group of seven acromegalic patients, treated with 20 mg Pegvisomant for at least 18 months, a small but significant increase in bone mineral density [188, 189]. If confirmed in a larger cohort these results raise interesting questions on the interaction of increased GH concentrations or even of the GH-receptor antagonist Pegvisomant with other receptors, that is, the prolactin receptor or so far unknown receptors that positively interact with bone formation.

**Quality of Life**

The IGF-1 concentration has been shown to be a poor indicator of complete clinical remission. Thus, to assess the clinical efficacy of Pegvisomant therapy Quality of Life (QoL) Questionnaires specifically developed for acromegalic patients have been used to monitor clinical efficacy of the medication. In a prospective, double blind, placebo-controlled, cross-over investigation QoL as well as symptoms scores were assessed with or without Pegvisomant in acromegalic patients with IGF-1 within normal limits. With Pegvisomant 40 mg/week QoL and symptoms scores improved. However, there was no correlation between the IGF-1 concentration and the improvement of QoL [190].

**Side Effects**

**Tumour Growth**

Due to the mechanism of action some safety considerations regarding possible tumour growth during Pegvisomant therapy had to be considered. In a large survey comprising 307 patients an increase in pituitary volume was reported in 18 (5.9%) of the patients [191]. Tumour growth was confirmed in 8 patients on reevaluation of the MRI. In three of these patients preceding long-term tumour growth could be demonstrated, irrespective of the therapy applied. In two patients tumour growth was most probably due to the reexpansion of the adenoma after withdrawal of SRL therapy before initiating Pegvisomant. In the remaining three patients minor tumour growth was diagnosed 14 months after initiation of Pegvisomant. The volume increase was considered clinically irrelevant and Pegvisomant therapy was continued. In six of the eight patients with tumour growth, the GH concentration could be determined by a specific assay excluding cross-reaction with Pegvisomant. All had an increase of the endogenous GH concentration above 60 ng/mL, while this was the case in only 44 (13.6%) of patients without tumour growth. Thus determination of the GH concentration during Pegvisomant with an assay that does not crossreact with the drug might identify those patients at risk for tumour growth. Overall, the risk of Pegvisomant-induced tumour growth was felt to be very low [191].

**Other Side Effects**

**Increase of Liver Enzymes**

Overall the drug is well tolerated and subjective complaints are few. An increase in liver enzymes has been observed and is reversible after ending treatment with Pegvisomant [192]. Small increases in transaminases (>3 times upper limit of normal, *N* = 20) normalised spontaneously in 10/20 patients despite ongoing therapy [193]. Cholestatic liver dysfunction, with a histological diagnosis of drug-related liver toxicity, developed in a patient with Gilbert syndrome and resolved after treatment was stopped [194]. Subsequently the authors surveyed 36 Spanish patients on Pegvisomant and found the UGT1A1*28 polymorphism associated with Gilbert syndrome to be a significant predictor of the risk of liver function abnormalities during Pegvisomant therapy [195]. Basal liver function tests should be performed and monitored monthly during the titration phase and repeated at longer intervals thereafter.

**Lipohypertrophy**

In the German Acrostudy 12 out of 371 patients reported some degree of lipohypertrophy [193]. An impressive form of lipohypertrophy has been illustrated by Buyuktas et al. in one patient [196]. Pathophysiologically the GH antagonism of Pegvisomant may reduce the lipolytic effect of GH and thus increase local fat deposition. Untreated acromegalic patients have a decreased fat mass due to the lipolytic effect of GH. Increased lipolysis causes insulin resistance and deterioration of glucose tolerance. If GH is normalised by surgery, the increased lipolysis will disappear and fat mass, insulin sensitivity, and GT will subsequently normalize. However, if GH is normalised by SRL therapy, the drug induced suppression of insulin secretion, directly and via suppression of GLP-1 [92], increases lipolysis. Hence, the decreased fat mass and glucose intolerance persist, but they are now drug-induced. In contrast, during Pegvisomant therapy, GH no longer stimulates lipolysis due to the blockade of its receptor, while insulin action is unabated. Therefore, insulin sensitivity should improve and fat mass, including intra-abdominal fat, should increase. This has been confirmed in five acromegalic patients on Pegvisomant. We demonstrated a significant increase of intra-abdominal fat by electron beam computer tomography, while the amount of subcutaneous abdominal fat remained unchanged [174]. Since intra-abdominal fat is a cardiovascular risk factor long-term follow-up is necessary to show whether detrimental complications will develop.

**Conclusions**

The GH-receptor antagonist Pegvisomant normalises IGF-1 concentration in up to 97% of the patients. The dose is titrated to normalize IGF-1 concentration, because GH cannot be routinely measured and serve as an indicator for therapeutic efficacy, due to cross-reactivity of the routinely used GH assay with Pegvisomant. Because of problems with the available IGF-1 assays the evaluation of therapy progress and efficacy may be difficult. It has been demonstrated that combined therapy with SRL is almost as effective as Pegvisomant monotherapy, but can be substantially more cost-effective. The drug is well tolerated and the risk for tumour regrowth during therapy seems to be low. The effect on adipose tissue and its consequences will have to be kept in mind. Liver function tests should be used to monitor therapy. However in most cases liver toxicity is reversible. Antibody development against Pegvisomant has been observed, their clinical relevance is unknown.

## 4. Overall Conclusions

Medical therapy of acromegaly is indicated in patients who failed to achieve the current remission criteria, that is, (i) a GH nadir below 1 *μ*g/L during a 75 g oral glucose load, (ii) a normal age- and sex-adjusted IGF-1 concentration either after surgery or following radiotherapy. In order to support therapeutic decisions a mean GH concentration >2.5 *μ*g/L and clear signs and symptoms of active acromegaly argue for further therapy. Therapeutic interventions should be orientated on those biochemical indices that indicate a safe GH/IGF-1 concentration, as data on complete remission or even cure of acromegaly are still highly controversial due to problems of assay technologies and standardisation.

Medical therapy is mostly initiated with SRL. However because of the high price of all SRL, dopamine agonists should be the initial therapeutic principle in patients with low basal GH/IGF-1 concentrations. Up to 10% will achieve remission with dopamine agonist monotherapy alone. If SRL insufficiently suppresses GH secretion dopamine agonists can be added, preferentially Cabergoline, and this will normalise GH secretion in up to 50% of these patients. If dopamine agonists fail to normalise GH secretion, combination therapy of SRL and Pegvisomant is recommended. Combination therapy rather than Pegvisomant monotherapy is preferred due to its higher cost-effectiveness. With these therapeutic strategies up to 95% of the patients will achieve complete remission (Figure 1).

## Figures and Tables

**Table 1:** Dosage and duration of action of different SRL.

Substance	Application	Dose range	Duration of action
Octreotide	Subcutaneous	150–300 (1500) *μ*g/d	*∼*6–8 hours
Octreotide LAR	Intramuscular	10–30 mg/month	*∼*4 weeks
Lanreotide SR	Intramuscular	10–20 mg/10–14 days	*∼*2 weeks
Lanreotide Autogel	Deep subcutaneous	60–120 mg/4–6 weeks	*∼*4–6 weeks

**Table 2:** Side-effects.

	Somatostatin analogues	Dopamine agonists	Pegvisomant
Often	Erythema at injection site	Nausea/vomiting	Headache
	Swelling at injection site	Headache	Vertigo
	Reduced appetite	Vertigo	Somnolence/asthenia
	Nausea/vomiting	Asthenia	Tremor
	Abdominal discomfort	Diarrhoea/constipation	Diarrhoea/constipation
	Bloating	Vasospasm	Nausea/vomiting/bloating
	Diarrhoea	Oedema	Sweating/pruritus
	Gallstones	Sleeping disorders	Hypercholesterolemia
	Reduced glucose tolerance		Hyperglycaemia
			Weight increase
			Hypertension
			Lipohypertrophy
			Abnormal liver function tests
			Pain at injection site

Rare	Alopecia	Orthostatic dysregulation	Thrombocytopenia
	Skin allergies	Psychomotoric disturbances	Leukocytopenia/leukocytosis
	Bradycardia	Visual hallucination	Hyperaesthesia
	Pancreatitis	Psychosis	Narcolepsy
	Reversible acute hepatitis	Retroperitoneal fibrosis	Visual disturbances
	Vitamin B12 deficiency	Valvular dysfunction	m. Meniere
	Abnormal liver function tests	Abnormal liver function tests	Dyspnoea
			Hematuria/proteinuria/polyuria
			Arthralgia/myalgia
			Hypertriglyceridaemia
			Hypoglycaemia
			Fever
			Sleeping disturbances

**Table 3:** Comparison of primary versus secondary medical therapy (mTx).

	*N*	Medication	Dose	Remission (% of pats)		Criteria for remission		Author
				GH	IGF-1	GH	IGF-1	
Prim. mTx	26	Octr. s.c.	777 *μ*g/d (mean)	43	68	<2 *μ*g/L	Normal**	Newman et al. 1998 [135]
Sec. mTx	81		635 *μ*g/d (mean)	22	62			
				*P* = ns	*P* = ns			

Prim. mTx	23	Lanr. SR	30 mg/10–14 d	64	51	basal GH < 7.5 U/L	Normal*	Baldelli et al. 2000 [136]
Sec. mTx	71			78	70			
				*P* < 0.05	*P* < 0.05			

Prim. mTx	15	Octr. LAR	10–40 mg/mo	73	53	<2.5 *μ*g/L or <1 *μ*g/L (oGTT)	Normal*	Colao et al. 2001 [119]
Sec. mTx	21			76	71			
				*P* = ns	*P* = ns			

Prim. mTx	10	Octr. LAR or Lanr. SR	20 mg/6 w–30 mg/4 w	50	60	<2 *μ*g/L	Normal*	Ayuk et al. 2002 [134]
Sec. + prim. mTx	22		30 mg/2 w–30 mg/10 d	36	62			

*normal IGF-1 concentration, age adjusted; **normal IGF-1 concentration, not age adjusted.
